# Novel insights into the differential functions of Notch ligands in vascular formation

**DOI:** 10.1186/2040-2384-1-8

**Published:** 2009-11-16

**Authors:** Tsutomu Kume

**Affiliations:** 1 Feinberg Cardiovascular Research Institute, Feinberg School of Medicine, Northwestern University, 303E Chicago Ave, Chicago, IL 60611, USA

## Abstract

The Notch signaling pathway is a critical component of vascular formation and morphogenesis in both development and disease. Compelling evidence indicates that Notch signaling is required for the induction of arterial-cell fate during development and for the selection of endothelial tip and stalk cells during sprouting angiogenesis. In mammals, two of the four Notch receptors (Notch1 and Notch4) and three of the five Notch ligands (Jagged1, Dll1, and Dll4) are predominantly expressed in vascular endothelial cells and are important for many aspects of vascular biology. During arterial cell-fate selection and angiogenesis, the roles of Notch1 and Notch4 are thought to be similar, and the function of Dll4 is well-characterized. However, the molecular mechanisms that determine the functional similarities and differences of Notch ligands in vascular endothelial cells remain largely unknown; consequently, additional research is needed to elucidate the ligand-specific functions and mechanisms associated with Notch activation in the vascular endothelium. Results from recent studies indicate that Dll1 and Dll4 have distinct roles in the specification and maintenance of arterial cell identity, while Dll4 and Jagged1 have opposing functions in tip- and stalk-cell selection during sprouting angiogenesis. This review will focus on the newly discovered, distinct functions of several Notch ligands in the regulation of blood vessel formation and will provide perspectives for future research in the field.

## Introduction

Notch signaling is evolutionarily conserved and critical for cell-fate determination, differentiation, and many other biological processes [1]. The mammalian Notch signaling pathway is composed of four Notch receptors (Notch1-4) and five ligands (Jagged1 and 2 and Delta-like [Dll] 1, 3, and 4). All of the ligands are transmembrane-type proteins and, consequently, Notch signaling is often mediated by cell-cell interactions. Transmission generally occurs between neighboring cells that express high levels of either the receptor or the ligand, although receptor-ligand coexpression occurs in some cells, such as vascular endothelial cells. Over the last decade, numerous studies have demonstrated that Notch signaling is critically involved in vascular development and disease [2-6]. For example, Notch signaling is required for arterial cell-fate determination during embryonic development, and the Notch pathway controls both developmental and pathological angiogenesis by modulating the selection of endothelial tip and stalk cells in newly sprouting blood vessels. Regulation of the Notch pathway in blood vessels has been well characterized; however, the specific roles of each Notch ligand during vascular formation and morphogenesis are unknown. Recent studies provide insight into the distinct functions of Notch ligands in blood vessels, and this review summarizes the current understanding of how several ligands differentially activate Notch signaling in the vasculature.

## Basic mechanisms of the Notch signaling pathway

**Figure 1:**
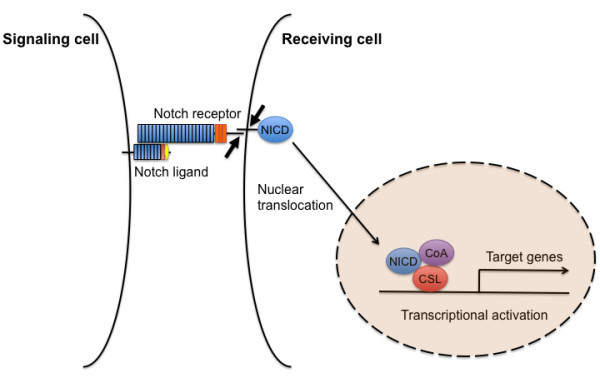
**A diagram of the canonical Notch signaling pathway**. This schematic shows a simplified overview of the main components of Notch signaling. Upon Notch ligand binding, a two-step proteolysis cleavage process (small arrows) within the juxtamembrane region and transmembrane domain of the Notch receptor is catalyzed by a member of the disintegrin and metalloproteases (ADAMS) family and the γ-secretase containing complex, respectively, then the Notch intracellular domain (NICD) is released from the membrane and translocates to the nucleus, where it forms a transcriptional activation complex with CSL and coactivators (CoA), thereby inducing the transcription of target genes.

Notch signaling is initiated by interactions between a Notch ligand expressed on the surface of one cell (the signaling cell) and a Notch receptor expressed on the surface of a neighboring cell (the receiving cell). Upon ligand binding, Notch is sequentially cleaved, and the Notch intracellular domain (NICD) is released into the cytoplasm. The NICD enters the nucleus, where it interacts with the transcription factor CSL (named after mammalian CBF1, *Drosophila *Su(H), and *Caenorhabditis elegans *LAG1) to form a transcriptional activation complex that induces expression of the bHLH transcription factors (Hes and Hey families) (Figure 1). This signaling mechanism is considered the "canonical" Notch pathway; non-canonical Notch signaling has also been reported [7].

**Figure 2:**
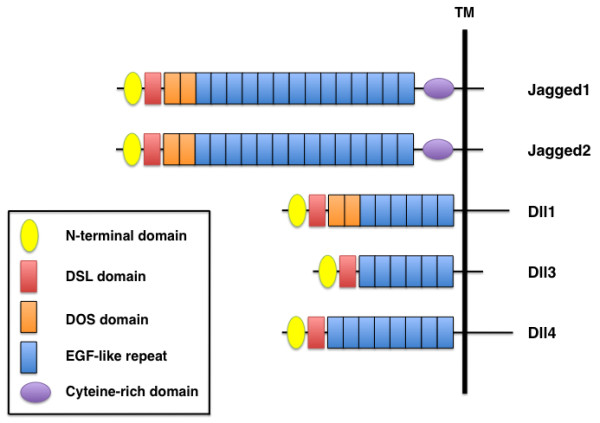
**Domain organization of mammalian Notch ligands**. Five mammalian ligands are classified into two categories, Delta-like (Dll1, Dll3, Dll4) and Serrate-like (Jagged1, Jagged2), based on structural homology to the two Drosophila ligands, Delta and Serrate. All Notch ligands have an N-terminal domain, a DSL (Delta/Serrate/LAG-2) domain and EGF-like repeats. Jagged1 and Jagged2 contain a cysteine-rich domain, whereas Jagged1, Jagged2, and Dll1 have two DOS (Delta and OSM-11-like proteins) domains located immediately following the DSL domain.

The extracellular domains of mammalian Notch ligands have several distinct features that participate in receptor binding (Figure 2). Their N-terminal regions contain a conserved module, and a second conserved module, the DSL (Delta/Serrate/LAG-2) domain, is located adjacent to the N-terminal region. Both Notch ligands and receptors contain multiple EGF-like repeats, and the ligands Jagged1, Jagged2, and Dll1 have tandem EGF repeats that form the DOS (Delta and OSM-11-like proteins) domain [8]. Jagged1 and Jagged2 also contain a cysteine-rich domain located between the EGF-like repeats and the transmembrane domain. Both the DSL and DOS domains are critical for receptor binding [9], and the structural diversity of Notch ligands is determined by the presence or absence of the cysteine-rich DOS domains.

Activation of Notch signaling through cell-cell interactions (*trans*-interactions) has been well characterized; however, Notch ligands also regulate the Notch pathway by binding to Notch receptors within the same cell (*cis*-interactions) [10,11]. In general, *trans*-interactions between Notch ligands and receptors activate Notch signaling, whereas *cis*-interactions are believed to inhibit Notch signaling [9]. The precise mechanisms that mediate Notch activation by the *cis*-interactions remain unclear, and further studies need to be performed [12].

## Notch receptor and ligand expression in blood vessels

**Figure 3:**
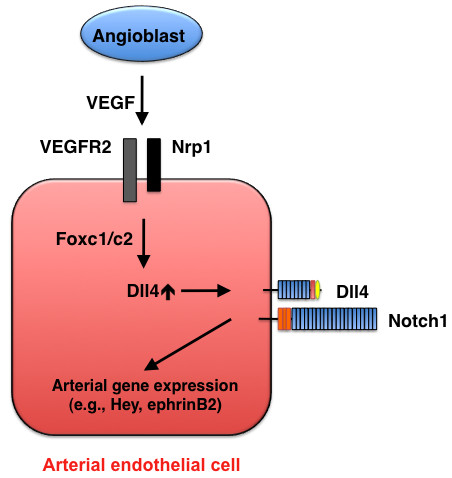
**Arterial cell specification mediated by Dll4-Notch signaling**. During early development, VEGF (in concert with Foxc1/c2 transcription factors) induces Dll4 expression in endothelial cells, and Dll4-Notch signaling promotes arterial gene expression.

**Figure 4:**
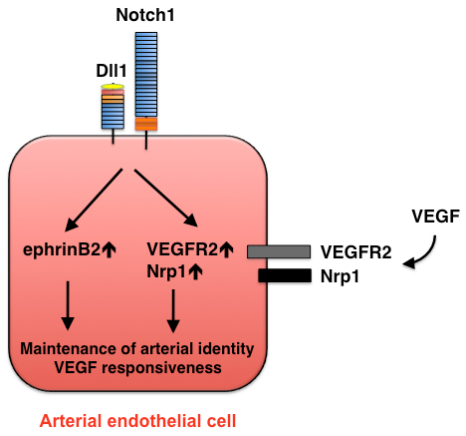
**Maintenance of arterial identity mediated by Dll1-Notch signaling**. At a later stage of development, Dll1 expression is induced in arterial endothelial cells and is required for maintenance of the arterial phenotype. Dll1 also acts upstream of VEGF by regulating the expression of VEGFR2 and its co-receptor neuropilin 1 (Nrp1).

**Figure 5:**
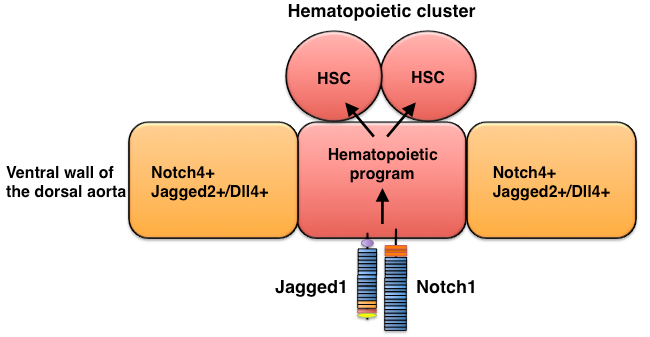
**Jagged1-mediated hematopoietic program in the dorsal aorta during development**. Hematopoietic stem cells (HSC) descend from Jagged1+ Notch1+ endothelial cells in the aorta.

**Figure 6:**
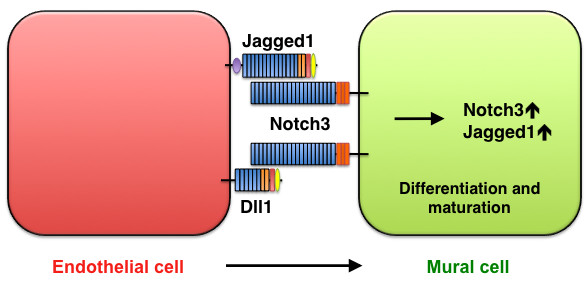
**Smooth-muscle maturation mediated by Jagged1/Dll1-Notch3 signaling**. Jagged1 and Dll1 in endothelial cells activate Notch3 on mural cells, thereby promoting mural-cell maturation.

**Figure 7:**
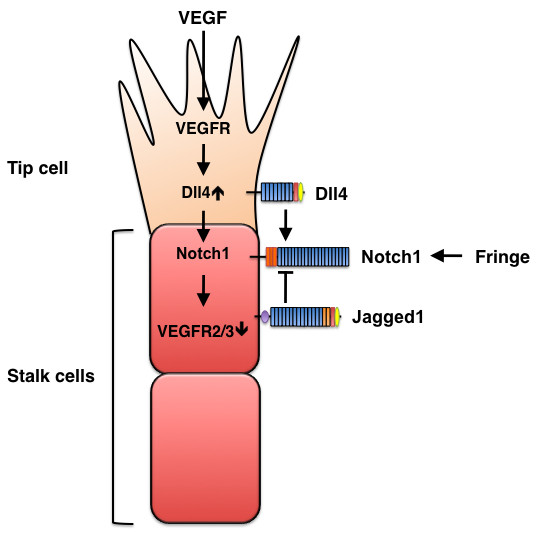
**Opposing effects of Dll4 and Jagged1 on sprouting angiogenesis**. VEGF signaling induces Dll4 expression in tip cells, and Dll4, in turn, activates Notch signaling in stalk cells, which reduces stalk-cell sensitivity to VEGF stimulation and, consequently suppresses the tip-cell phenotype. Conversely, Jagged1 antagonizes Dll4-mediated Notch activation in stalk cells to increase tip cell numbers and enhances vessel sprouting. The antagonistic effects of the two ligands are controlled by Fringe-dependent modulation of Notch signaling.

Notch1 is broadly expressed in many tissues, including the heart and vascular endothelial cells, while Notch4 expression is restricted to vascular endothelial cells [13-15], and Notch3 is predominantly expressed in vascular smooth muscle cells [16]. Transcriptional regulation of *Notch4 *in vascular endothelial cells is controlled by fibroblast growth factor 2 (FGF2), the signal-dependent transcription-factor activator protein 1 (AP-1), and the glucocorticoid receptor [14,15,17]. Four of the five known mammalian Notch ligands (Jagged1, Jagged2, Dll1, and Dll4) are expressed in vascular endothelial cells [13,18-20]; Jagged1 is also expressed in smooth muscle cells surrounding the arteries and plays an important role in smooth muscle cell maturation [21]. The molecular mechanisms that control the expression of Notch ligands in vascular endothelial cells and smooth muscle cells have been frequent topics of recent research (Table 1). For example, Dll4 expression during arterial specification and for tip-cell selection during vessel sprouting [22-28] is mediated by vascular endothelial growth factor (VEGF). Importantly, and as described below, the expression patterns of the Notch ligands vary both spatially and temporally and, consequently, the localization of each ligand is likely to be critical for Notch activation in blood vessels (Figures 3, 4, 5, 6, and 7).

**Table 1:** Signaling pathways/factors that regulate Notch ligand expression in vascular endothelial and smooth muscle cells

Pathway/factor	Ligand	Cell type	Biological effect	References
VEGF	Dll4 (↑)	Endothelial cells	Arterial specification	[22,24,27]
		Endothelial tip cell formation in sprouting angiogenesis		[23,25,26,28]
TNFα	Dll4 (↓) Jagged1 (↑)	Endothelial cells	Tip cell and stalk cell selection	[52]
VEGF + FGF2 (synergistic)	Dll1 (↑)	Endothelial cells	Ischemia-induced postnatal arteriogenesis	[38]
PDGF/angiotensin II	Jagged1 (↓)	Smooth muscle cells	Growth regulation	[45]
Jagged1 (endothelial)	Jagged1 (↑)	Mural cells	Smooth muscle cell maturation	[21]

↑, Upregulation; ↓, Downregulation.

VEGF, vascular endothelial growth factor; TNF, tumor necrosis factor; FGF, Fibroblast growth factor; PDGF, platelet-derived growth factor

## Notch1, Notch4, and the ligands Dll1 and Dll4 during arterial specification and maintenance

Results from recent studies in zebrafish suggest that activation of Notch signaling by the Sonic hedgehog (Shh) and VEGF pathways is essential for arterial specification during development [29,30]. Two Notch receptors, Notch1 and Notch4, are predominantly expressed in arterial endothelial cells of early mouse embryos. *Notch1 *mutant mice die with cardiovascular defects during early development [13], and endothelial-specific ablation of *Notch1 *in mice leads to embryonic lethality and vascular abnormalities that are associated with angiogenesis [31]. An endothelial-specific *Notch1*+/- mutation has also been associated with impaired postnatal neovascularization in a marine hind-limb ischemia model [32]. These results demonstrate the cell-type specific role of Notch1 in the vascular endothelium during development and postnatal life (Table 2). *Notch4 *mutant mice display no conspicuous phenotype, but the vascular defects observed in compound *Notch1; Notch4 *mutant embryos are more severe than those in *Notch1 *mutants [13], which suggests that the functions of Notch1 and Notch4 overlap during vascular development. Endothelial expression of a constitutively active Notch4 mutant from the *Flk1 *(*VEGFR2*) locus results in embryonic vascular abnormalities such as disorganized vascular networks and dilated blood vessels [33], and in adult mice, constitutively active Notch4 expression has been associated with arteriovenous malformations (AVMs) (i.e., abnormal connections between arteries and veins) that are accompanied by ectopic expression of the arterial marker ephrinB2 in veins [34]. Furthermore, the impaired arterial and venous differentiation associated with constitutively active Notch4 expression can be reversed by suppressing Notch4 activity in the endothelium [34]. Collectively, these findings suggest that both reduced and elevated Notch signaling can lead to vascular abnormalities and, consequently, that the maintenance of proper Notch signaling is critical during vascular development.

**Table 2:** Mammalian Notch receptors and ligands involved in vascular development and disease

Component	Phenotype/Role	References
**Notch receptors**		
Notch1	Proper vascular development; Postnatal neovascularization	[13,31,32]
Notch3	Maturation of vascular smooth muscle cells	[44]
Notch4	Null mice show normal vascular development; *Notch1; Notch4 *mutant mice have severe vascular defects; Gain-of-function experiments show vascular abnormalities in development and postnatal life	[13,33,34]
**Notch ligands**		
Jagged1	Dispensable for arterial specification; Formation of hematopoietic stem cells from the aorta; Smooth muscle differentiation and maturation; Proangiogenic regulation	[21,39-41,52]
Dll1	Maintenance of arterial identity; Arterial smooth muscle differentiation; Postnatal arteriogenesis	[19,38]
Dll4	Arterial specification; Tip cell and stalk cell selection during sprouting angiogenesis; Regulation of tumor angiogenesis	[23,25,35-37,46-52,55,56]

Of the four Notch ligands (Jagged1, Jagged2, Dll1, and Dll4) that are expressed in arterial endothelial cells, Dll4 alone is expressed in the dorsal aorta of mice at embryonic day 8.5 (E8.5), and its expression is restricted to vascular endothelial cells [13]; thus, Dll4 is believed to be the ligand for Notch1 and Notch4 during early vascular development (Figure 3). *Dll4 *mutant mice display early embryonic lethality with impaired arterial specification and AVMs that appear in a genotype-dependent manner (i.e., the severity increases with the number of mutant alleles) [35-37]. These observations further emphasize the importance of maintaining proper Notch activity levels during vascular development. Foxc1 and Foxc2 transcription factors directly activate the *Dll4 *promoter in endothelial cells, and their induction of *Dll4 *expression is enhanced by VEGF, which suggests that Foxc1 and Foxc2 act upstream of Notch signaling during arterial-cell specification [22,27].

Dll1 expression is detected in arterial endothelial cells at a later stage (E13.5) of mouse development [19] and continues to be restricted to arterial endothelial cells in adults [38]. Dll1 is not critically involved in arterial-cell specification; however, analyses in hypomorphic and endothelial-specific *Dll1 *mutant mice indicate that Dll1 is required for the maintenance of arterial identity [19]. Expression of the arterial marker ephrinB2 is reduced, and the venous marker COUP-TFII is upregulated, in endothelial-specific *Dll1 *mutant mice, despite Dll4 expression in the mutant endothelial cells [19]; thus, Dll4 appears to be essential for initiating the arterial program, whereas Dll1 is required to maintain arterial identity during embryonic development. In addition, Sorensen et al. have shown that Dll1-mediated Notch1 activation upregulates VEGF receptor 2 (VEGFR2) and its coreceptor, neuropilin-1, which suggests that Dll1 enhances the responsiveness of arterial endothelial cells to VEGF signaling. Thus, Dll4-mediated Notch signaling occurs downstream of VEGF during arterial specification, whereas Dll1-mediated Notch signaling acts upstream of VEGF to maintain arterial identity (Figures 3 and 4). Dll1 is also important for ischemia-induced postnatal arteriogenesis and the induction of ephrinB2 [38].

Jagged1 does not play a critical role in arterial development [39-41] but is required for the definitive hematopoietic program in the dorsal aorta. After arterial and venous endothelial cells differentiate, the ventral region of the dorsal aorta, located in the aorta-gonad-mesonephros (AGM) region of the mid-gestation mouse embryo (around E10-11), generates the first adult hematopoietic stem cells (HSCs). Notch4 is broadly expressed throughout the aortic endothelium of the AGM, whereas Notch1 expression is restricted to the ventral region of the dorsal aorta [40,42]. Importantly, three Notch ligands (Jagged1, Jagged2, and Dll4) have distinctive expression patterns in the dorsal aorta of the AGM: Jagged1 and Notch1 expression overlap in the dorsal aorta, Jagged2 expression occurs in endothelial cells adjacent to Notch1-positive endothelial cells, and Dll4 is expressed in both Notch1-positive and Notch1-negative endothelial cells [40,42]. Analyses in *Jagged1 *mutant mice indicate that Jagged1 is required for Notch1 activation during the induction of intra-embryonic definitive hematopoiesis in the AGM [40]. Jagged2 and Dll4 expression in the AGM of *Jagged1 *mutant embryos is normal, and hematopoiesis is normal in *Jagged2 *mutant mice [40]; thus, the function of Jagged1 is distinct from Dll4 and Jagged2 activity during the hematopoietic program of the newly formed aorta (Figure 5).

## Notch3, Jagged1, and Dll1 during smooth-muscle differentiation and maturation

Notch3 is predominantly expressed in the vascular smooth muscle of arteries and is not expressed in veins. Mutations in human *NOTCH3 *are associated with cerebral autosomal dominant arteriopathy with subcortical infarcts and leukoencephalopathy (CADASIL), a disorder that causes stroke and dementia and is accompanied by the degeneration of vascular smooth muscle cells [43]; adult *Notch3 *mutant mice display a defect in the maturation of arterial smooth muscle cells [44]. As noted above, *Jagged1 *mutant mice exhibit normal arterial development [39,40], yet endothelial-specific *Jagged1 *mutants have impaired vascular smooth muscle differentiation [39]. This observation indicates that Jagged1 expression in the arterial endothelium activates Notch in neighboring cells, and that this function is critical for smooth muscle cell differentiation. Jagged1 expression by endothelial cells induces mural cells (pericytes in the microvasculature or smooth muscle cells in larger vessels) to express Notch3 and Jagged1, which subsequently promotes and maintains the differentiation phenotype of mural cells [21], whereas platelet-derived growth factor (PDGF) and angiotensin II downregulate Notch3 and Jagged1 expression in vascular smooth muscle cells [45]. Furthermore, a recent study found that expression of the arterial smooth muscle marker smoothelin is impaired in *Dll1 *mutant mice [19], and this decline has also been observed in *Notch3*-mutant arteries [44]. Taken together, these findings suggest that Jagged1 and Dll1 are the primary ligands that regulate Notch3 activity during smooth-muscle differentiation and maturation (Figure 6).

## Dll4 and Jagged1 in tip- and stalk-cell specification during sprouting angiogenesis

The formation of new blood vessels, a process known as angiogenesis, involves the sprouting of endothelial cells. In response to VEGF stimulation, filopodia extend from a migratory endothelial cell at the vessel's tip (i.e., the tip cell), and proliferative endothelial cells (i.e., stalk cells) form the trunk of the new vessel. Recent studies in mice and zebrafish clearly demonstrate that Notch signaling interacts with VEGF signaling during tip-cell and stalk-cell specification [5]. VEGF induces Dll4 expression in tip cells, then Dll4 activates the Notch pathway in adjacent endothelial cells to reduce expression of VEGFR2 and VEGFR3, thereby suppressing the tip-cell phenotype, and tip-cell phenotype suppression cell-autonomously promotes the stalk-cell phenotype. Together, these mechanisms balance tip-cell and stalk-cell selection and, consequently, limit the number of sprouting vessels (Figure 7). Genetic or pharmacological disruption of Dll4-Notch signaling leads to excessive tip-cell formation and vessel sprouting in cultured cells, in zebrafish and mouse embryos, and during tumor angiogenesis [23,25,46-51].

By using endothelial-specific *Jagged1 *mutant mice and mice that overexpress *Jagged1 *in vascular endothelial cells, Benedito et al. demonstrated that Jagged1 enhances angiogenesis and antagonizes the effects of Dll4-mediated Notch signaling during sprouting angiogenesis [52]. Jagged1 is strongly expressed in stalk cells, whereas Dll4 is predominantly detected in tip cells [52], and the antagonistic interaction between Dll4 and Jagged1 in endothelial cells is mediated by the glycosyltransferase Fringe, which regulates the posttranslational modifications of Notch receptors in a ligand-dependent manner. Fringe enhances Notch activation in response to Delta-like ligands and reduces Notch activity in response to Jagged ligands [12]; consequently, Fringe increases Dll4-induced endothelial Notch signaling and reduces Notch signaling in response to Jagged1 [52]. Jagged1 also appears to promote vascular sprouting by regulating VEGFR3 expression in tip cells [52]. Taken together, these results illustrate the opposing effects of Dll4 and Jagged1 on sprouting angiogenesis.

## Notch ligands in pathological angiogenesis

Dll4 is expressed in tumor vasculature [26,36,53,54], and as observed in studies of developmental angiogenesis, the blockade of Dll4-mediated Notch signaling (via systemic administration of Dll4-neutralizing antibodies [47,48] and systemic or local administration of modified Dll4 proteins [47,55]) increased tumor-vessel sprouting, which indicates that Dll4-Notch signaling is critical for tip- and stalk-cell selection during tumor angiogenesis. Remarkably, the inhibition of Dll4-Notch signaling increased neovascularization but impaired tumor growth, because the non-productive angiogenesis reduced tumor perfusion. Conversely, Dll4 activation of endothelial Notch signaling reduces tumor angiogenesis, but increases vessel diameter and perfusion, which enhances tumor growth [47,56]. For these reasons, Dll4 is now recognized as a potential therapeutic target for tumor angiogenesis [57].

As described above, Jagged1 antagonizes Dll4 during sprouting angiogenesis [52], and overexpression of Jagged1 in tumor cells has been shown to enhance neovascularization and tumor growth [58]; however, the role of Jagged1 in pathological angiogenesis (including tumor angiogenesis) is not yet fully understood. Current findings suggest that angiogenic sprouting in the tumor is tightly controlled by positive and negative regulation of Jagged1 and Dll4 in both endothelial and non-endothelial cells. Recent studies have shown that a soluble form of Notch1 (Notch decoy) acts as an antagonist of ligand-dependent Notch signaling by (potentially) interfering with Dll1, Dll4, and Jagged1 [59,60]. Importantly, the Notch decoy reduces tumor growth without increasing vessel growth, which suggests that the effects of the Notch decoy differ from those induced by Dll4 blockade. It is therefore likely that the proangiogenic function of Jagged1 in tumor cells and endothelial cells could also influence tumor angiogenesis.

## Notch signaling in peripheral ischemia

Notch signaling is also required for angiogenesis in peripheral ischemia models [32,38] (Table 2). Blood flow recovery and postnatal neovascularization in response to hind-limb ischemia are impaired in both global and endothelial-specific *Notch1*+/- mice, but not in *Notch4*-/- mice [32]. Dll1 is strongly induced in arterial endothelial cells during ischemia-induced arteriogenesis, and *Dll1*+/- mice display reduced collateral-artery growth and impaired blood-flow recovery after hind-limb ischemia [38]. Notch activation and ephrinB2 induction are not observed in the collateral arteries of *Dll1*+/- mice [38].

## Concluding remarks and future perspectives

Studies performed in the past few years clearly demonstrate that the different Notch ligands have distinct functions in vascular development and disease. This understanding has prompted numerous investigations into the mechanisms by which Notch signaling is essential for multiple aspects of vascular biology. However, given that the effects of Notch pathway activation on endothelial cells are context-dependent [4], many questions remain to be answered. First, the upstream signaling pathways that control the expression of Notch ligands in blood vessels remain largely unknown; VEGF induces Dll4 expression in endothelial cells (Table 1), but Jagged1 is absent in tip cells where Dll4 is highly expressed, which suggests that the two ligands are regulated differently. Second, the selective activation of Notch in vascular endothelium remains unclear; for example, Notch signaling is not activated in arteries of *Dll1 *mutant mice, despite the presence of Jagged1 and Dll4 [19]. Third, the role of non-canonical Notch ligands, such as microfibril-associated glycoprotein (MAGP)-2 [9], is poorly understood. MAGP-2 binds to Jagged1, Jagged2, Dll1, and Notch1 [61,62], and is known to modulate Notch signaling in sprouting angiogenesis [63,64], but the mechanistic basis for the function of MAGP-2 in ligand-dependent Notch activation has yet to be elucidated. Finally, given that Dll4 and Jagged1 have opposing effects on angiogenesis, experiments that specifically inhibit each ligand with selective neutralizing antibodies [65] may be important not only for understanding how Notch is activated in the vasculature, but also for the development of therapeutic strategies designed to control angiogenesis by targeting Notch signaling.

## Competing interests

The author declares that he has no competing interests.

## Authors' contributions

The author drafted and wrote the manuscript.

## Author's information

The author is an Associate Professor at Northwestern University School of Medicine, USA. He completed his postdoctoral training in the lab of Brigid Hogan at the Howard Hughes Medical Institute at Vanderbilt University Medical Center, USA. He graduated with a Ph.D. in Molecular and Cellular Biology from the University of Tokyo, Japan.
